# The trophoblast plug during early pregnancy: a deeper insight

**DOI:** 10.1007/s00418-016-1474-z

**Published:** 2016-08-10

**Authors:** Gregor Weiss, Monika Sundl, Andreas Glasner, Berthold Huppertz, Gerit Moser

**Affiliations:** 1 Institute of Cell Biology, Histology and Embryology, Medical University of Graz, Harrachgasse 21/7, 8010 Graz, Austria; 2 Femina Med Center, Herrengasse 9, 8010 Graz, Austria

**Keywords:** Placenta, Spiral artery, Trophoblast plug, Endovascular trophoblast, Endoglandular trophoblast, Uterine gland

## Abstract

During the first trimester of pregnancy, foetal endovascular trophoblasts invade into maternal spiral arteries, accumulate and form plugs in the lumen of the vessels. These plugs only allow blood plasma to seep through. Hence, during the first trimester of pregnancy, a first flow of fluids through the placental intervillous space is established, resulting in a physiological oxygen gradient between mother and foetus. The trophoblast plugs block spiral arteries until the beginning of the second trimester (11–14 weeks). In parallel, uterine glands are invaded and opened by endoglandular trophoblasts towards the intervillous space of the placenta, without showing the formation of plugs (Moser et al. in Hum Reprod 25:1127–1136, 2010, Hum Reprod Oxf Engl 30:2747–2757, 2015). This enables histiotrophic nutrition of the embryo prior to onset of maternal blood flow into the placenta. Failure of these endovascular and endoglandular invasion processes may lead to miscarriage or pregnancy disorders such as intrauterine growth restriction (IUGR). After dissolution of the plugs, the onset of maternal blood flow allows maternal blood cells to enter the intervillous space and oxygen concentrations rise up. In this study, we demonstrate for the first time serial cross sections through a trophoblast plug in a first trimester placental bed specimen. Invaded and plugged arteries as well as invaded uterine glands in week 11 of gestation are visualized with specific immunohistochemical double staining techniques. We show that spiral artery plugs appear throughout the placental invasion zone and illustrate erythrocytes stowed due to trophoblast plugs. In addition, we give evidence of the presence of MMP-1 in plugs of invaded spiral arteries. The results reveal a better understanding and a closer insight into the morphological appearance of trophoblast plugs and the consequences for placental and uterine blood flow.

## Introduction

Trophoblast invasion is a crucial event in human pregnancy and especially during the first trimester of pregnancy. The trophoblast subpopulations, villous and extravillous trophoblast, originate from cytotrophoblast stem cells (Bischof and Irminger-Finger 2005). In normal, uncomplicated pregnancies extravillous trophoblasts (EVTs) invade through the uterine interstitium towards decidua and myometrium (*interstitial trophoblast*). The invasion process is linked to appropriate cell to cell contact of EVT with the surrounding tissues, conducted by numerous cytokines like IL6 and uPAR expressed at the foetal–maternal interface (Weiss et al. 2016).

Prior to trophoblast invasion, the walls of spiral arteries undergo a variety of reorganization processes including widening of the vessel lumen (Brettner 1964), swelling of smooth muscle cells (Craven et al. 1998) and vacuolation of endothelial cells (Boyd and Hamilton 1967). During the invasion period, a subpopulation of EVT, *endovascular trophoblasts*, invades from the uterine interstitium into maternal spiral arteries and subsequently lines and remodels them. They penetrate through the endothelium and reach the lumen of the vessel where they accumulate and form trophoblast plugs (Huppertz et al. 2014; Kaufmann et al. 2003). These trophoblast plugs block the blood flow from the mother towards the placenta during the first trimester and finally disintegrate at the end of the first trimester, allowing maternal blood flow towards the intervillous space of the placenta (Jauniaux et al. 2000).

Besides invasion into spiral arteries, trophoblasts also invade towards uterine glands (*endoglandular trophoblast*). They attach, erode and replace the glandular epithelium, thus opening the uterine glands towards the intervillous space and thereby ensuring histiotrophic nutrition of the embryo prior to the onset of maternal blood flow (Moser et al. 2010, 2015).

In this study, we demonstrate the presence of trophoblast plugs and their progression within the placental bed tissues in immunohistochemically stained serial cross sections for the first time. We show that spiral artery plugs appear throughout the placental invasion zone and illustrate erythrocytes stowed due to trophoblast plugs. Furthermore we were able to detect MMP-1 in trophoblast plugs and thereby confirming the invasive phenotype of the endovascular trophoblasts.

## Materials and methods

### Tissue collection and processing

First trimester placental tissue (week 7–12 of gestation, *n* = 40) was obtained from elective termination of pregnancy. Informed consent was obtained with approval of the ethical committee of the Medical University of Graz. The tissue was rinsed in Hank’s buffered salt solution (HBSS, Gibco Life Technologies, Austria), supplemented with 1 % penicillin/streptomycin and 1 % amphotericin B (Gibco Life Technologies). Tissue was subsequently collected for fixation in 4 % formalin for 24 h and embedded in paraffin.

After embedding, serial 5 µm sections were cut and placed on Superfrost Plus slides (Menzel, Braunschweig, Germany). For subsequent staining, sections were deparaffinized in xylene and rehydrated through a series of graded alcohol. Heat-induced antigen retrieval was performed in antigen retrieval solution at pH 9 (Leica Biosystems, Nussloch, Germany) in a pressure cooker (Model DC2002, Biocare Medical, Concord, USA) for 7 min at 120 °C before immunohistochemistry.

### Immunohistochemistry

Immunohistochemistry was performed using the UltraVision LP Detection system (Thermo Scientific, Fremont, USA) according to the manufacturer’s instructions. Primary antibodies were diluted in antibody diluent (Dako, Vienna). Table 1 lists details of all antibodies and the appropriate immunoglobulin G (IgG) negative control antibodies used in their respective dilutions. Sections were counterstained with Mayer’s haemalaun and mounted with Kaiser’s glycerol gelatine (Merck, Vienna Austria).

**Table 1:** Antibodies used in immunohistochemistry

Antigen/antibody	Company	Dilution	Host/isotype
Cytokeratin 7 (KRT7)	Acris (Herford, Germany)	1:1000	Rabbit IgG pc
Major histocompatibility complex, class I, G (HLA-G)	BD Biosciences (Vienna, Austria)	1:1000	Mouse IgG mc
Matrix metalloproteinase 1 (MMP-1)	Protein Tech (Rosemont, USA)	1:500	Rabbit IgG pc
Von Willebrand factor (VWF)	Sigma Aldrich (St. Louis, USA)	1:1000	Rabbit IgG mc
Mouse IgG1 (DAK-GO1)	Dako (Carpinteria, USA)	1:100	Mouse IgG mc
Rabbit immunoglobulin fraction (X 0903)	Dako (Carpinteria, USA)	1:300	Rabbit IgG mc

### Immunohistochemical double staining

Immunohistochemical double labelling was performed using the MultiVision Polymer Detection system (MultiVision anti-rabbit/AP + anti-mouse/HRP polymers; Thermo scientific, Fremont, USA) according to the manufacturer’s instructions. Primary antibodies were diluted in antibody diluent (Dako). Binding of primary antibodies was visualized with the chromogens of the MultiVision kit, termed “LVBlue” and “LVRed”.

One specimen (week 11 of gestation) showed outstanding plug structures. This specimen was examined in detail.

### Microscopic evaluation

**Fig. 1:**
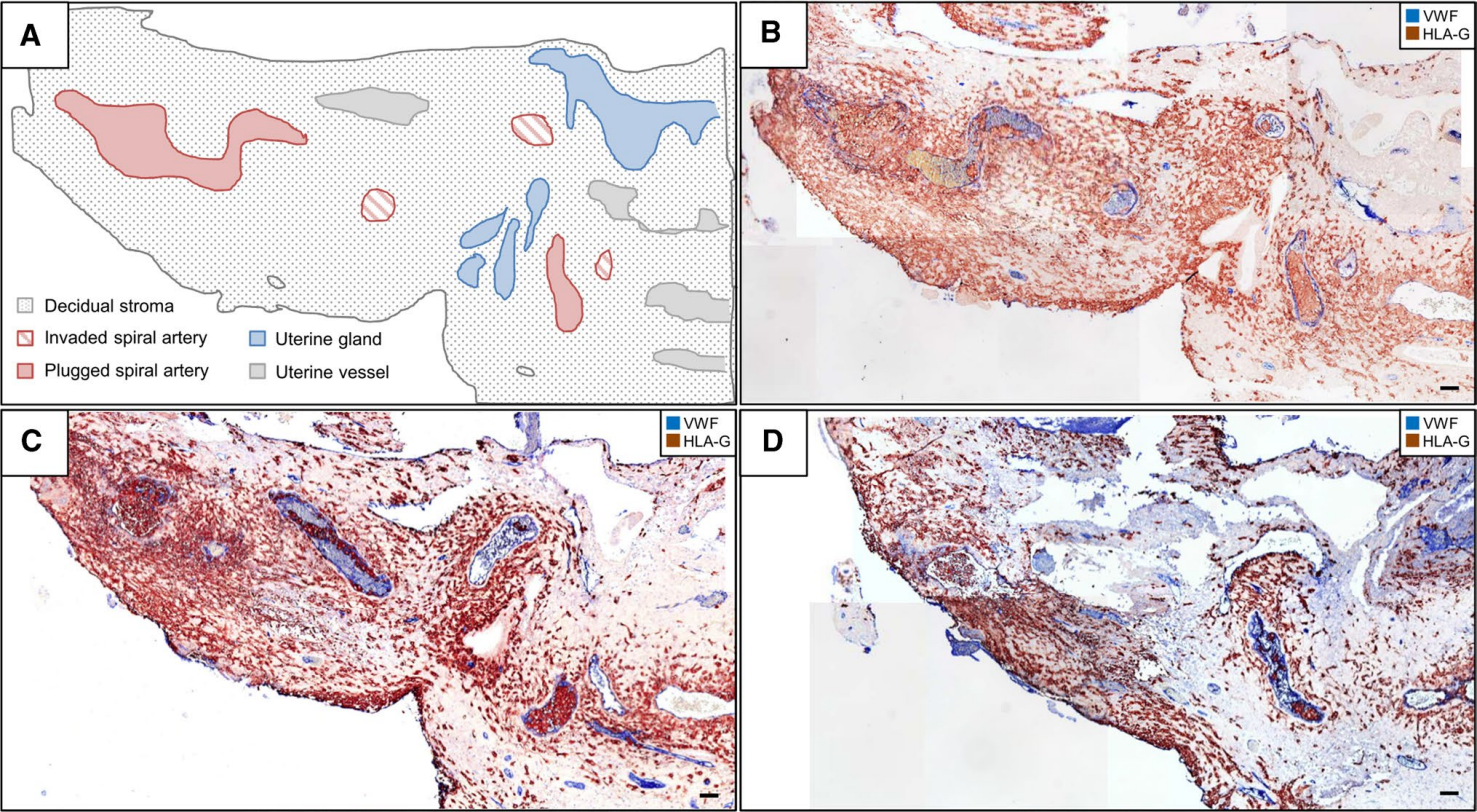
Trophoblast invasion and arterial trophoblast plugs—overview. Immunohistochemical double staining of serial cross sections of invaded decidua basalis (gestational age 11 weeks) for major histocompatibility complex, class I, G (HLA-G) (*brown*, serves as marker for extravillous trophoblasts) and von Willebrand factor (vWF) (*blue*, serves as marker for vascular endothelial cells). No nuclear counterstain. **a** Schematic outline of structures of interest within the cross section in (**b**). Decidual stroma is *dotted*, plugged arteries are marked *red*, invaded arteries are marked with *red stripes*. Uterine glands are marked in *blue* and uterine vessels in *grey*. **b** Corresponding histological cross section to the scheme in image **a**. Various invaded and plugged sectors of spiral arteries can be identified within the section. **c** Progression of the section in (**b**) with a distance of 55 µm. Alterations of plugs and invaded vessels and glands (specifically identified in respective stainings of serial sections, not shown) are present. **d** Further changes of invaded structure with a distance of another 60 µm. *Scale bar* represents 50 µm

**Fig. 2:**
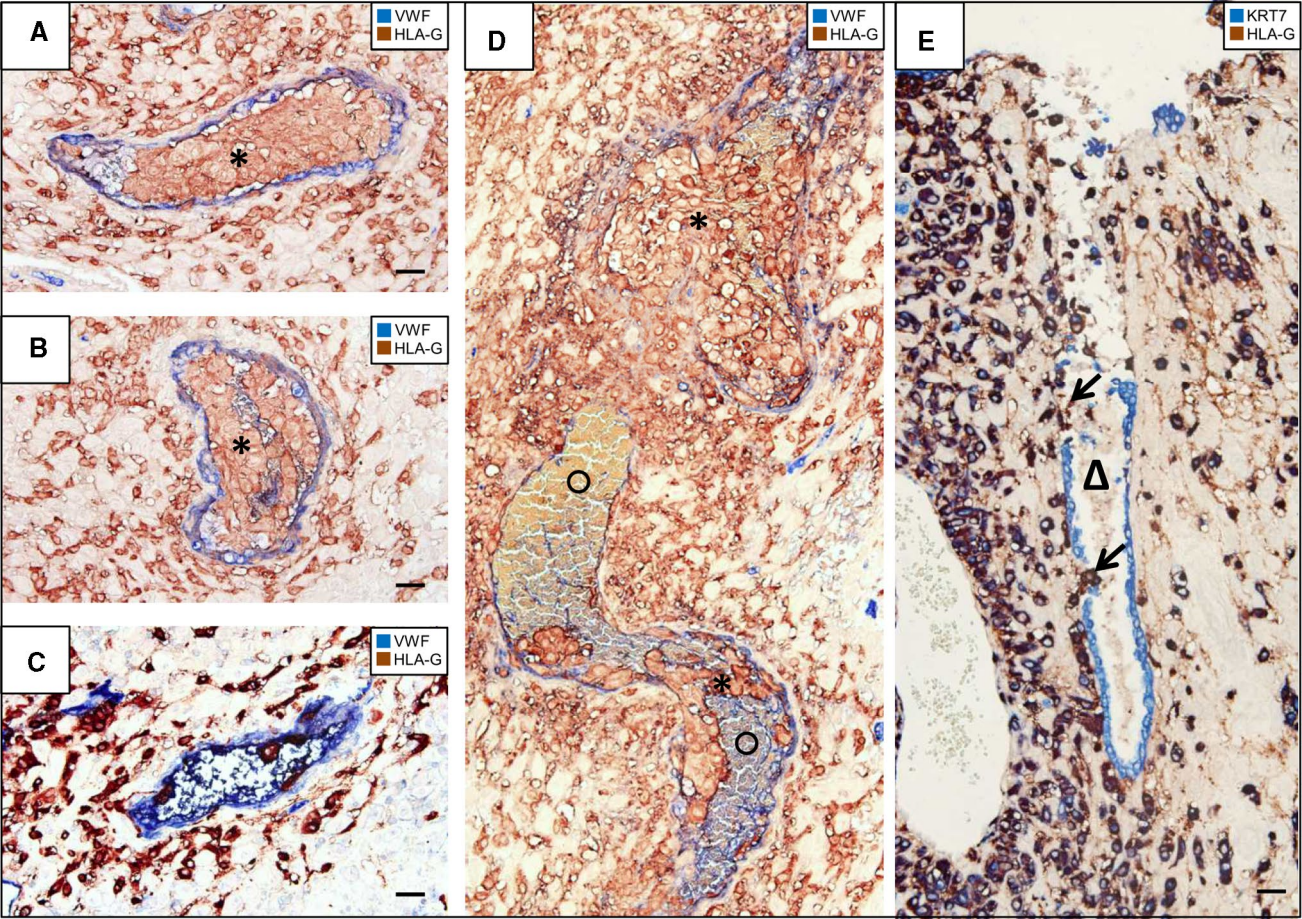
Arterial trophoblast plugs and invasion of uterine glands—details. Immunohistochemical double staining of serial cross sections of invaded decidua basalis (gestational age 11 weeks) for major histocompatibility complex, class I, G (HLA-G) (*brown*, serves as marker for extravillous trophoblasts) and von Willebrand factor (vWF) (*blue*, serves as marker for vascular endothelial cells) (**a**–**d**) or cytokeratin 7 (KRT7) (*blue*, serves as marker for glandular epithelium here) (**e**). No nuclear counterstain. (**a**–**c**) Progression of a trophoblast plug (*asterisk*) in consecutive cross sections: In (**a**) and (**b**) the trophoblast plug fills the complete lumen of the spiral artery, whereas in (**c**) there are only few endovascular trophoblasts left. (**b**) is in 55 µm distance to (**a**) and in 60 µm distance to (**c**). (**d**) highlights the most prominent plugged artery in the current placental specimen. Erythrocytes (*circle*) appearing *yellow-greenish-blue* are stowed by the trophoblast plug (*asterisk*). (**e**) demonstrates invasion of endoglandular trophoblasts (*arrows*) into a uterine gland (*triangle*). The gland is opened towards the intervillous space. *Scale bar* 50 µm

A microscope (model DM6000B; Leica) equipped with a motorized stage and a digital camera (model DP72; Olympus Austria GmbH, Vienna, Austria) were used for image acquisition. Images from serial sections were taken in different magnifications (50×–200×) and were manually stitched together with Microsoft Power Point 201 (Images in Figs. 1b–d, 2d, e).

One trophoblastic plug was followed in 35 consecutive sections until the plug was no longer visible. In 9 out of these slides, single trophoblasts were counted manually with an average of 58 cells per plug cross section. According to this number, we extrapolated a minimum number of extravillous trophoblasts within the plug.

## Results

### Invasion and plugging of uterine spiral arteries in the first trimester of pregnancy

We examined serial sections of decidua basalis containing trophoblast plugs. Due to the number of serial sections (5 µm in length) of the specimen, we examined a total of 120 µm of the respective plug area.

In the progression of the sections, extravillous trophoblast invasion and trophoblast plug(s) could be tracked and followed through the tissue.

For a better understanding and overview, Fig. 1a highlights the most prominent structures in the present placental bed section including invaded and plugged spiral arteries as well as uterine glands and vessels. Figure 1b shows the cross section reproduced in Fig. 1a. Immunohistochemical double staining of vWF and HLA-G shows two trophoblast plugs and invaded spiral artery structures within the placental tissue in this section. vWF also binds to fibrinoid structures, which is the reason for the blue staining inside all blood vessels. Furthermore uterine gland structures are partly invaded by extravillous trophoblast cells. Isotype-negative control antibodies did not reveal any staining (data not shown).

Figure 1c shows the consecutive region in another section in a distance of 55 µm. Parts of the big plug on the left side of the image can still be seen, and another segment of the big plug rises in the middle of the picture, where also uterine glands are strongly invaded by trophoblast cells. The trophoblast plug on the right side of the image shows another cut of the plug, smaller in diameter but still tightly packed with trophoblasts (Fig. 1c).

Another 60 µm further, we can only presume the presence of the big plug, while the one on the right side shows elongation in diameter, indicating another segment of a spiral artery, partly filled with trophoblasts.

In Fig. 2a–c, we demonstrate the progress of one plug on the right side of the sections in Fig. 1 in more detail. Figure 2a shows the plug tightly filled with trophoblasts. In a distance of 55 µm, the plug diameter changes and some erythrocytes in the lumen of the plug can be visualized (Fig. 2b). In Fig. 2c, we see the plug another 60 µm apart, showing just a few trophoblasts left in the lumen of the vessel and more erythrocytes indicating the start of trophoblast infiltration upstream of the respective vessel.

Figure 2d shows a higher magnification of the big plug on the left side in Fig. 1. The longitudinal section of the spiral artery reveals the presence of the trophoblast plug. Erythrocytes (appear yellow-greenish) are stowed by the plug, and further trophoblasts are invading the vessel.

Besides spiral arteries also uterine glands are invaded by extravillous trophoblast cells (endoglandular trophoblast). Figure 2e shows a uterine gland next to a uterine vessel. The glandular epithelial cells are partly dissolved and replaced by trophoblast cells, opening the gland towards the intervillous space.

### MMP-1 presence in trophoblast plugs

**Fig. 3:**
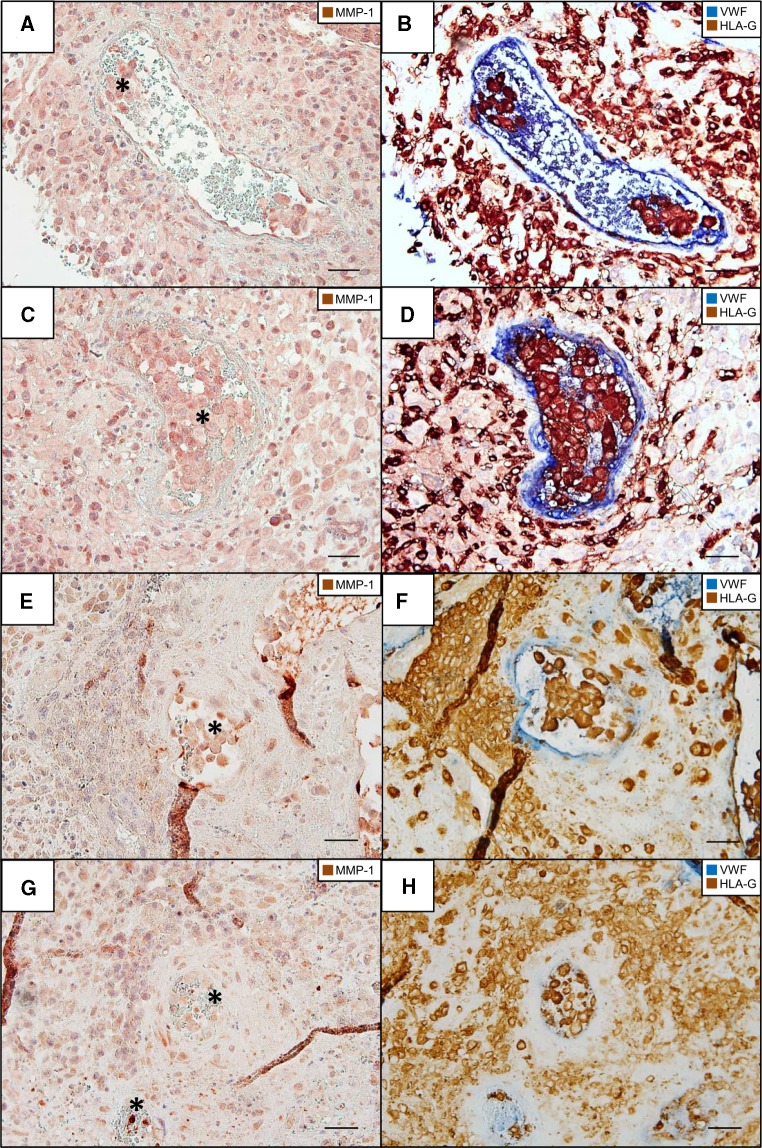
MMP-1 expression in arterial trophoblast plugs. Immunohistochemical single (**a**, **c**, **e**, **g**) and double (**b**, **d**, **f**, **h**) staining of serial sections (**a**–**b**, **c**–**d**, **e**–**f**, **g**–**h**) of invaded decidua basalis (gestational age 11 weeks **a**–**d** and gestational week 7 **e**–**h**) for matrix metalloproteinase 1 (MMP-1) and for major histocompatibility complex, class I, G (HLA-G) (*brown*, serves as marker for extravillous trophoblasts) together with von Willebrand factor (vWF) (*blue*, serves as marker for vascular endothelial cells). Nuclei are counterstained with Haemalum (**a**, **c**, **e**, **g**) or no nuclear counterstain (**b**, **d**, **f**, **h**). **a**, **c**, **e**, **g** Endovascular trophoblasts within trophoblast plugs (*asterisk*) are clearly positive for MMP-1. Additionally interstitial trophoblasts as well as decidual stroma cells are positive for MMP-1. **b**, **d**, **f**, **h** Immunohistochemical double staining in serial sections enables a clear classification of the assessed structures and cells and clearly confirms the extravillous origin of the trophoblasts composing the trophoblast plug. *Scale bar* 50 µm

To elucidate the presence of MMP-1 in extravillous trophoblasts in plugged spiral arteries, we performed immunohistochemistry in serial sections of the trophoblast plug. Figure 3a, c demonstrates the presence of MMP-1 in trophoblast plugs of spiral arteries and invading trophoblasts, respectively (week 11 of gestation). Additionally, serial sections were immunohistochemical double stained with antibodies against vWF and HLAG (Fig. 3b, d, f, h). The plugs show a clear expression of MMP-1, potentially higher than invading trophoblasts in the surrounding tissue. Also, specimen in week 7 of gestation show clear MMP-1 staining in trophoblast plugs (Fig. 3e, g). Isotype-negative control antibodies did not reveal any staining (data not shown).

### Counting of trophoblast cells in a plug structure

We counted the trophoblast cells present in the plugs from the serial sections and roughly estimated that this trophoblast plug is composed of at least 720 single endovascular trophoblasts and extends over at least 115 µm.

## Discussion

In the present study, we were able to demonstrate that trophoblast plugs block spiral arteries at the end of the first trimester (week 11 of gestation) by assessing serial sections of placental bed specimens with specific immunohistochemical single and double staining. In parallel, uterine glands were invaded and opened towards the intervillous space of the placenta by endoglandular trophoblasts, without showing the formation of plugs. Furthermore, the presence of MMP-1 in endovascular trophoblast within trophoblast plugs was verified.

During the first trimester, endovascular trophoblasts invade into the lumen of spiral arteries and form trophoblast plugs. These plugs only allow blood plasma to seep through to reach the intervillous space. Hence, a first flow of fluids through the placental intervillous space is established, resulting in a physiological oxygen gradient between mother and placenta and the foetus (Huppertz et al. J Reprod Immunol 2014). This oxygen gradient plays a central role in differentiation and invasion of extravillous trophoblasts. Growth and development of the embryo and the placenta are strongly influenced by the appropriate oxygen environment and changes of oxygenation throughout pregnancy (Huppertz et al. 2014). The physiological and therefore normoxic oxygen concentration for placenta and embryo until the end of the first trimester of pregnancy are below 20 mmHg barometric column (Jauniaux et al. 2000; Rodesch et al. 1992). Elevated oxygen levels during this period may cause pregnancy complications and spontaneous abortion (Burton and Jauniaux 2004; Jauniaux et al. 2003).

Trophoblast plugs block the spiral arteries until the beginning of the second trimester (11–14 weeks) (Huppertz et al. 2014). For the first time, we show and visualize that erythrocytes (and putatively other blood cells) are stowed by the trophoblast plug, only allowing maternal blood plasma to seep through. This indicates the importance of the plugs to prevent the onset of placental blood flow prior to the end of the first trimester.

After dissolution of the plugs, the onset of maternal blood flow allows maternal blood cells to enter the intervillous space and oxygen concentrations rise up. The oxygen gradient disappears (Huppertz et al. 2009, 2014).

Starting from interstitial trophoblast invasion, EVTs do not only invade into uterine spiral arteries (*endovascular trophoblast*), another side branch of EVT invades into uterine glands (*endoglandular trophoblasts*), which are spread throughout the decidua during the first trimester of pregnancy (Moser et al. 2010). Until recently, it was commonly believed that the role of uterine glands is restricted to the process of implantation.

However, there is increasing evidence that they continue delivering nutritional components like proteins, carbohydrates and lipids and also immunosuppressive factors like MUC-1 into the intervillous space of the placenta post-implantation (Amoroso 1952; Brayman et al. 2004; Burton et al. 2007). The replacement of uterine glandular epithelial cells by endoglandular trophoblasts and the opening of uterine glands towards the intervillous space ensure histiotrophic nutrition of the embryo prior to onset of maternal blood flow during the first trimester (Moser et al. 2010). It has not yet been shown that failure in the replacement of uterine glands results in pregnancy pathologies, but it is tempting to speculate that early spontaneous miscarriages may also result from missing nutritional support following a failure of invasion of uterine glands.

In line with the findings of Moser et al. (2010), we were able to demonstrate invasion of extravillous trophoblasts into uterine glands and the aperture of these glands towards the intervillous space (Moser et al. 2015). We did not observe plug formation in glandular structures, which is not surprising due to the fact that histiotrophic nutrition of the embryo would be distorted by plugging the glands prior to the onset of maternal blood flow at the end of the first trimester.

It is commonly known that cytokines play essential roles in the process of trophoblast invasion. Numerous studies have shown the importance of cytokines released from trophoblasts most prominently cytokines like IL-8, IL-13 and RANTES (Naruse et al. 2010). Lately, also cytokines like GRO, IL6 and uPAR released from uterine arteries and veins have been linked to appropriate trophoblast invasion (Weiss et al. 2016). It is tempting to speculate that a strong interaction of cytokines released from trophoblasts on the one hand and cells of the invaded tissue on the other hand assures appropriate invasion of the tissue.

MMP-1, in particular, is known to be a prominent invasion marker. The proteinase degrades collagen (Hulboy et al. 1997) and enables trophoblast cells to migrate through the uterine tissue. Deficient activity has been associated with the pregnancy pathologies like pre-eclampsia and intrauterine growth restriction (IUGR).

MMP-1 is commonly expressed in cells of the maternal–foetal interface, including EVT (Huppertz et al. 1998), cytotrophoblasts, syncytiotrophoblast (Vettraino et al. 1996) and decidual cells (Lockwood et al. 2014). It has been reported that decidual endothelial cells express a low amount of MMP-1 in pre-eclamptic decidual tissue (Gallery et al. 1999). So far, the presence of invasion markers like MMP-1 in trophoblasts of endovascular plugs has only been proved in the rhesus monkey (Blankenship and Enders 1997). Here, we demonstrate the presence of MMP-1 also in human trophoblast plugs, indicating the invasive potential of endovascular trophoblasts and supporting the thesis that endovascular trophoblasts originate from interstitial trophoblasts (concept of intravasation) (Kaufmann et al. 2003).

**Fig. 4:**
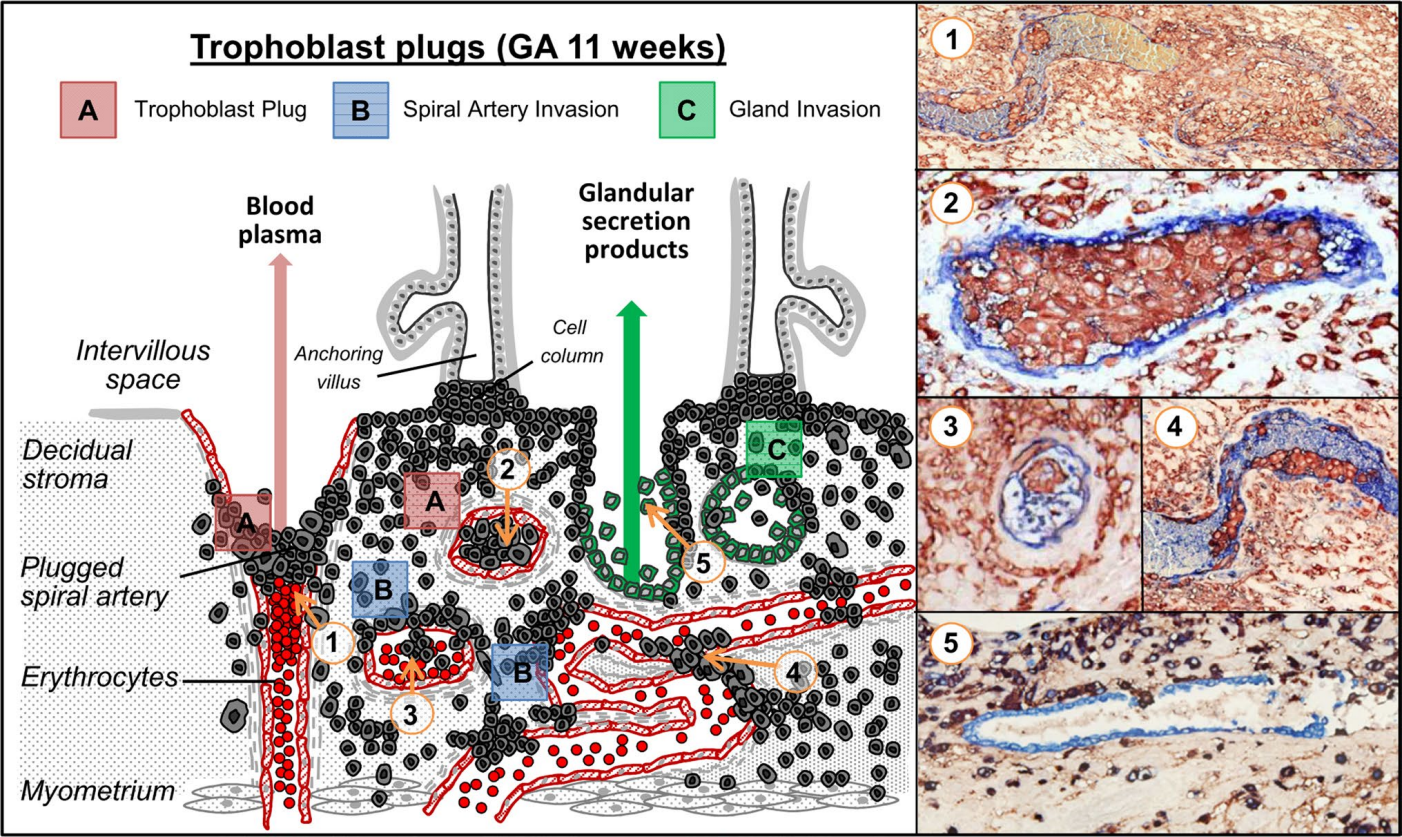
Different perspectives of trophoblast plugs within spiral arteries. Trophoblast plugs (*A*) are composed of endovascular trophoblast cells. Depending on the stage of invasion and on the localization of sectioning the lumen of the spiral artery can be either completely filled with trophoblasts (*1*, *2*) or partly infiltrated (*3*, *4*). In a plugged artery, the approaching erythrocytes are stowed by the trophoblast plug (*1*). Uterine glands are invaded and opened towards the intervillous space (*C*) by endoglandular trophoblasts. Glandular epithelial cells are replaced by endoglandular trophoblasts (*5*)

The scheme in Fig. 4 demonstrates the routes of trophoblast invasion and summarizes the different perspectives of trophoblast plugs with the corresponding in situ images (Fig. 4). In conclusion, the results reveal a better understanding and a closer insight into the morphological details of trophoblast plugs and the consequences for placental and uterine blood flow. In future studies, it will be interesting to gain more knowledge of characteristics of endovascular trophoblasts within the trophoblast plugs.

## References

[CR1] Amoroso EP (1952) Placentation. In: Parkes AS (ed) Marshalls physiology of reproduction. Longmans Green and Co., London, pp 127–311

[CR2] Bischof P, Irminger-Finger I (2005). The human cytotrophoblastic cell, a mononuclear chameleon. Int J Biochem Cell Biol.

[CR3] Blankenship TN, Enders AC (1997). Trophoblast cell-mediated modifications to uterine spiral arteries during early gestation in the macaque. Acta Anat (Basel).

[CR4] Boyd JD, Hamilton WJ (1967). Development and structure of the human placenta from the end of the 3rd month of gestation. J Obstet Gynaecol Br Commonw.

[CR5] Brayman M, Thathiah A, Carson DD (2004). MUC1: a multifunctional cell surface component of reproductive tissue epithelia. Reprod Biol Endocrinol.

[CR6] Brettner A (1964). On the behavior of the secondary wall of uteroplacental blood vessels during decidual reactions. Acta Anat (Basel).

[CR7] Burton GJ, Jauniaux E (2004). Placental oxidative stress: from miscarriage to preeclampsia. J Soc Gynecol Investig.

[CR8] Burton GJ, Jauniaux E, Charnock-Jones DS (2007). Human early placental development: potential roles of the endometrial glands. Placenta.

[CR9] Craven CM, Morgan T, Ward K (1998). Decidual spiral artery remodelling begins before cellular interaction with cytotrophoblasts. Placenta.

[CR10] Gallery ED, Campbell S, Arkell J, Nguyen M, Jackson CJ (1999). Preeclamptic decidual microvascular endothelial cells express lower levels of matrix metalloproteinase-1 than normals. Microvasc Res.

[CR11] Hulboy DL, Rudolph LA, Matrisian LM (1997). Matrix metalloproteinases as mediators of reproductive function. Mol Hum Reprod.

[CR12] Huppertz B, Kertschanska S, Demir AY, Frank HG, Kaufmann P (1998). Immunohistochemistry of matrix metalloproteinases (MMP), their substrates, and their inhibitors (TIMP) during trophoblast invasion in the human placenta. Cell Tissue Res.

[CR13] Huppertz B, Gauster M, Orendi K, Konig J, Moser G (2009). Oxygen as modulator of trophoblast invasion. J Anat.

[CR14] Huppertz B, Weiss G, Moser G (2014). Trophoblast invasion and oxygenation of the placenta: measurements versus presumptions. J Reprod Immunol.

[CR15] Jauniaux E, Watson AL, Hempstock J, Bao YP, Skepper JN, Burton GJ (2000). Onset of maternal arterial blood flow and placental oxidative stress. A possible factor in human early pregnancy failure. Am J Pathol.

[CR16] Jauniaux E, Hempstock J, Greenwold N, Burton GJ (2003). Trophoblastic oxidative stress in relation to temporal and regional differences in maternal placental blood flow in normal and abnormal early pregnancies. Am J Pathol.

[CR17] Kaufmann P, Black S, Huppertz B (2003). Endovascular trophoblast invasion: implications for the pathogenesis of intrauterine growth retardation and preeclampsia. Biol Reprod.

[CR18] Lockwood CJ, Basar M, Kayisli UA, Guzeloglu-Kayisli O, Murk W, Wang J, De Paz N, Shapiro JP, Masch RJ, Semerci N, Huang SJ, Schatz F (2014). Interferon-gamma protects first-trimester decidual cells against aberrant matrix metalloproteinases 1, 3, and 9 expression in preeclampsia. Am J Pathol.

[CR19] Moser G, Gauster M, Orendi K, Glasner A, Theuerkauf R, Huppertz B (2010). Endoglandular trophoblast, an alternative route of trophoblast invasion? Analysis with novel confrontation co-culture models. Hum Reprod.

[CR20] Moser G, Weiss G, Gauster M, Sundl M, Huppertz B (2015). Evidence from the very beginning: endoglandular trophoblasts penetrate and replace uterine glands in situ and in vitro. Hum Reprod Oxf Engl.

[CR21] Naruse K, Innes BA, Bulmer JN, Robson SC, Searle RF, Lash GE (2010). Secretion of cytokines by villous cytotrophoblast and extravillous trophoblast in the first trimester of human pregnancy. J Reprod Immunol.

[CR22] Rodesch F, Simon P, Donner C, Jauniaux E (1992). Oxygen measurements in endometrial and trophoblastic tissues during early pregnancy. Obstet Gynecol.

[CR23] Vettraino IM, Roby J, Tolley T, Parks WC (1996). Collagenase-I, stromelysin-I, and matrilysin are expressed within the placenta during multiple stages of human pregnancy. Placenta.

[CR24] Weiss G, Huppertz B, Siwetz M, Lang I, Moser G (2016). Arterial endothelial cytokines guide extravillous trophoblast invasion towards spiral arteries; an in vitro study with the trophoblast cell line ACH-3P and female non-uterine endothelial cells. Placenta.

